# Association between fish intake and incidence of sarcopenia in community-dwelling older adults after a 6-year follow-up: the Korean frailty and aging cohort study

**DOI:** 10.3389/fnut.2025.1543290

**Published:** 2025-01-28

**Authors:** Seunghyun Yi, Miji Kim, Chang Won Won, Yongsoon Park

**Affiliations:** ^1^Department of Food and Nutrition, Hanyang University, Seoul, Republic of Korea; ^2^Department of Health Sciences and Technology, College of Medicine, Kyung Hee University, Seoul, Republic of Korea; ^3^Department of Family Medicine, College of Medicine, Kyung Hee University, Seoul, Republic of Korea

**Keywords:** sarcopenia, oily fish, n-3 polyunsaturated fatty acids, seafood, usual gait speed, community-dwelling older adults

## Abstract

Previous studies have suggested beneficial effects of n-3 polyunsaturated fatty acids on sarcopenia. However, the associations of dietary fish intake with the prevalence of sarcopenia are inconsistent, and those with the incidence of sarcopenia has not been studied. This study investigated the hypothesis that seafood and fish consumption is inversely associated with the subsequent incidence of sarcopenia. Using data from the Korean Frailty and Aging Cohort Study, 503 non-sarcopenic community-dwelling Korean adults aged 70–84 years were followed-up for 6 years. Sarcopenia was defined according to the Asian Working Group for Sarcopenia 2019 consensus. Dietary intake was assessed using two non-consecutive 24-h dietary recalls at baseline. The incidence of sarcopenia was 37.8% after the 6-year follow-up. The intake of oily fish was inversely associated with the incidence of sarcopenia (OR 0.99; 95% CI 0.98–1.00; *p* for trend = 0.046) and that of low gait speed (OR 0.98; 95% CI 0.97–1.00; *p* for trend = 0.016) after the 6-year follow-up, adjusting for confounding factors. Consumption of total seafood, fish, non-oily fish, or shellfish was not significantly associated with the incidence of sarcopenia or its parameters, such as muscle mass, handgrip strength, usual gait speed, 5-times sit-to-stand test, or the Short Physical Performance Battery. The findings demonstrate that the consumption of oily fish could be beneficial in preventing sarcopenia, particularly by improving usual gait speed in Korean community-dwelling older adults, suggesting oily fish as a strategy to reduce sarcopenia risk.

## 1 Introduction

Sarcopenia is a widespread geriatric condition characterized by progressive and systemic loss of skeletal muscle mass, strength, and function. Sarcopenia leads to adverse outcomes, such as falls, physical disability, frailty, and mortality (1). An estimated 10–16% of older adults suffer from sarcopenia globally, which is expected to increase significantly in the coming decades (2). Among the risk factors contributing to the development of sarcopenia, age-related chronic inflammation, commonly referred to as “inflammaging,” is increasingly recognized as being crucial in the pathogenesis of sarcopenia (3).

N-3 polyunsaturated fatty acids (PUFAs) are particularly abundant in seafood and fish. Their anti-inflammatory properties highlight their potential dietary role in the development and management of sarcopenia (3). Cross-sectional studies among older Chinese individuals have reported that the intake of seafood or fish is not associated with the prevalence of sarcopenia (4–6). However, oily fish intake is positively associated with muscle strength (7, 8) and physical performance (9) in older Europeans. Additionally, n-3 PUFA intake was reported to be inversely associated with the prevalence of sarcopenia in older Australian men (10), Japanese individuals with type 2 diabetes (11), and Brazilians who received a kidney transplant (12), and positively associated with physical performance in older Finnish women (13), Australian individuals with subjective memory complaints (14), and older British adults (15). The ratio of n-3 PUFA to energy intake was also inversely associated with the prevalence of sarcopenic obesity in older Korean women based on data from the Korean National Health and Nutrition Examination Survey (KNHANES) (16).

Blood levels of n-3 PUFA, an objective indicator of fish consumption, have been inversely associated with the prevalence of sarcopenia in older Koreans (17) and Japanese patients with liver cirrhosis and hepatocellular carcinoma (18), and positively associated with physical performance in older Europeans (15, 19, 20), Koreans (21), and Japanese (22). Meta-analyses of clinical trials showed that supplementation of n-3 PUFA can enhance muscle mass, muscle strength, and physical performance in older adults (23, 24). N-3 PUFA promoted muscle protein synthesis by activating the mammalian target of rapamycin (mTOR) signaling pathway and prevented muscle degradation by downregulating inflammatory cytokines, thereby mitigating inflammation-induced muscle loss in immobilized aged rodent models (25). In addition, n-3 PUFA enhanced muscle strength by improving membrane fluidity and modulating neuromuscular transmission (25).

To the best of our knowledge, no previous study has investigated the relationship between the incidence of sarcopenia and n-3 PUFA. Therefore, the aim of the present study was to investigate the hypothesis that the consumption of seafood and fish is inversely associated with the incidence of sarcopenia in community-dwelling older Koreans after a 6-year follow-up.

## 2 Methods

### 2.1 Participants

**Figure 1:**
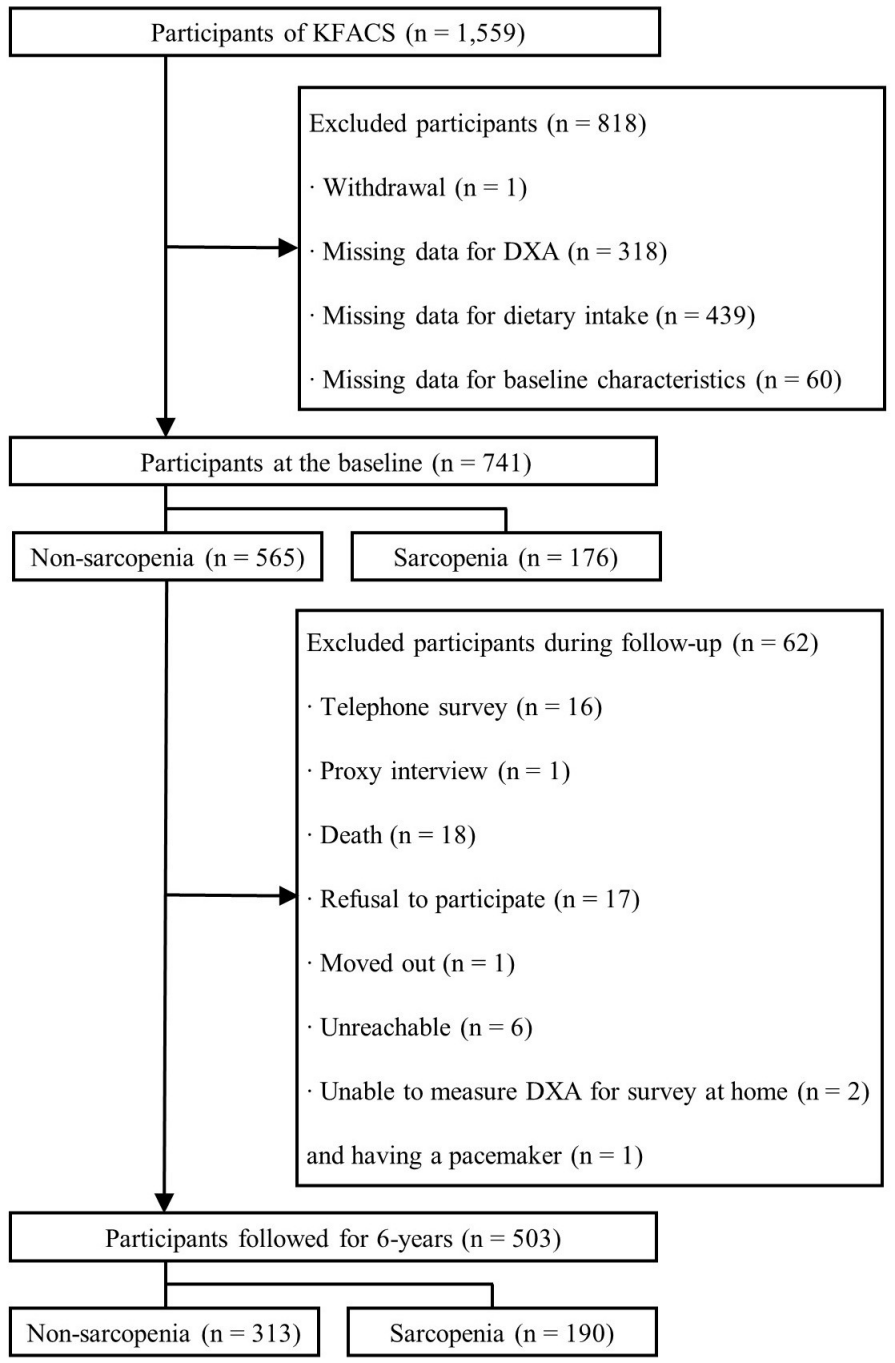
Flowchart of the participants for the 6-year follow-up. KFACS, Korean Frailty and Aging Cohort Study; DXA, dual-energy X-ray absorptiometry.

This longitudinal study was based on data from the Korean Frailty and Aging Cohort Study (KFACS), a nationwide multicenter cohort study of community-dwelling older adults aged 70–84 years (26). Surveys were performed across 10 centers, including eight medical hospitals and two public health centers in both urban and rural areas (Figure 1). In 2016, 1,559 participants were recruited, and dietary data and sarcopenia-related parameters were measured. At the baseline, 741 participants were included after exclusion owing to withdrawal of consent (*n* = 1), missing data for dual-energy X-ray absorptiometry (DXA) (*n* = 318), dietary intake (*n* = 439), educational level (*n* = 4), economic status (*n* = 6), comorbid status (*n* = 33), polypharmacy (*n* = 7), physical activity (*n* = 6), and sleep duration (*n* = 4). A total of 565 non-sarcopenic participants were followed up for 6 years in 2018, 2020, and 2022. At each follow-up period, sarcopenia-related parameters were measured to determine the incidence of sarcopenia, and non-sarcopenic participant diagnosed with sarcopenia was not followed up anymore. During follow-up, 62 participants were excluded since sarcopenia-related parameters were not measured at least once during any of the three follow-up assessments and thus sarcopenia status was unable to determine. The reasons for not determining sarcopenia were telephone survey (*n* = 16), proxy interview (*n* = 1), death (*n* = 18), refusal to participate (*n* = 17), relocation from the study area (*n* = 1), non-reachability (*n* = 6), inability to measure DXA for the survey at home (*n* = 2), or having a pacemaker (*n* = 1). Thus, 503 non-sarcopenic participants at the baseline were included in the final analysis. The present study was approved by the concerned Institutional Review Boards (KHUH-2015-12-103-107, 2021-05-081-039, and HYUIRB-202407-031), and written informed consent was obtained from all participants.

### 2.2 Assessment of sarcopenia

According to the 2019 consensus of the Asian Working Group for Sarcopenia (AWGS) (27), sarcopenia is defined as a low muscle mass with low muscle strength or physical performance. Appendicular skeletal muscle mass (ASM), calculated as the sum of the lean masses of both the arms and legs, was measured by DXA using either a Hologic DXA device (Hologic Inc., Bedford, MA, USA) or Lunar device (GE Healthcare, Madison, WI, USA). The ASM index was defined as ASM divided by height squared (kg/m^2^). Cut-off values for low muscle mass were ASM index < 7.0 kg/m^2^ for men and < 5.4 kg/m^2^ for women. Muscle strength was determined by measuring handgrip strength using a model TTK-5401 digital grip strength dynamometer (Takei Ltd., Tokyo, Japan). Each hand was alternately tested twice in the standing position and the highest value among the four grip strength measurements was obtained. The cut-off values for low muscle strength were < 28 kg for men and < 18 kg for women. Physical performance was assessed using the usual gait speed (UGS), 5-times sit-to-stand (5-STS) test, and Short Physical Performance Battery (SPPB). UGS was measured using a Gaitspeedmeter automatic gait speed timer (Dynamic Physiology, Daejeon, Korea). The participants were instructed to walk 4 m, with acceleration and deceleration phases of 1.5 m each. The average result of the two trials was used for the analysis. The 5-STS test measures the time taken to stand up five times from a sitting position, without depending on the chair arm. Participants were asked to perform the test as quickly as possible. The SPPB consists of standing balance tests (side-by-side, semi-tandem, and fully tandem), 5-STS test, and UGS assessment. Each SPPB test was scored from 0 to 4, with possible total scores ranging from 0 to 12 (28). Low physical performance was defined as meeting any of these three cut-offs: UGS < 1.0 m/s, 5-STS test ≥ 12 s, and SPPB score ≤ 9 for both sexes.

### 2.3 Dietary assessment

Dietary data were obtained from trained interviewers using the 24-h dietary recall method twice, in spring and fall, at baseline. The mean values were used for analysis. The portion size of food was estimated using bowls and plates, as well as food pictures developed by the National Institute of Health (NIH) and the Korea Disease Control and Prevention Agency (KDCA). Total energy and seafood intakes were calculated using the NIH and KDCA dietary assessment systems based on the database of the National Rural Living Science Institute (29). Seafood consumption was defined as the combined intakes of fish and shellfish. Based on the Composition Table of Marine Products in Korea 2018 by the National Institute of Fisheries Science (30), fish includes raw fish, canned fish, fish paste, salted fish, and shellfish, including oysters, clams, mussels, shrimp, lobster, and crayfish. According to the UK Scientific Advisory Committee on Nutrition (SACN), oily fish are defined as any species that contains 5–20% fat (31). In the present study, 16 types of fishes, such as mackerel, anchovies, salmon, sardines, and eels were classified as oily fish.

### 2.4 Covariates

Data on age, sex, smoking status, years of education, economic status, living arrangements, and history of falls and fractures during the previous year were collected. Weight and height were measured to the nearest 0.1 kg and 0.1 cm, respectively. Body mass index (BMI) was calculated as weight (kg) divided by height squared (m^2^). Low economic status refers to recipients of the National Basic Livelihood Security System or Medical Beneficial System (26). Comorbidities that were quantified included hypertension, dyslipidemia, myocardial infarction, congestive heart failure, angina, peripheral vascular disease, cerebrovascular disease, arthritis, osteoporosis, chronic obstructive pulmonary disease, asthma, diabetes mellitus, cancer, renal disease, and depression. Polypharmacy was defined as the use of five or more prescribed drugs in the previous 3 months (26). Sleep duration was categorized as < 7, 7–8, or > 8 h according to the National Sleep Foundation's sleep duration recommendations for older adults (32). Low physical activity was measured using the International Physical Activity Questionnaire to calculate the energy expenditure, defined as ≤ 494.65 kcal/week for men and ≤ 283.50 kcal/week for women (26). Cognitive impairment was assessed using a Korean Mini-Mental State Examination (K-MMSE) score < 24 (26), and nutritional status was evaluated using the Korean version of the Mini-Nutritional Assessment Short Form; a score ranging between 12 and 14 was defined to indicate normal nutritional status, that of 8–11 was associated with a risk of malnutrition, and < 7 points indicated malnutrition (26).

### 2.5 Statistical analyses

The Kolmogorov–Smirnov test was used to confirm the normal distribution of the variables. Continuous variables were presented as mean ± standard deviation (SD). Statistical significance was verified using the independent *t*-test for parametric variables and the Mann–Whitney test for non-parametric variables. The proportions of categorical variables are presented as the number of participants and percentages using the chi-square test. In the multivariate models, covariates with a *p*-value < 0.20 were selected as confounding factors (33). Age, BMI, smoking status, living arrangements, number of comorbidities, cognitive impairment, fall experience, and sleep duration were included in the fully adjusted model. The associations between the intake of seafood, fish, and shellfish and the incidence of sarcopenia, as well as each sarcopenia diagnostic criterion, were assessed with odds ratios (ORs) and 95% confidence intervals (CIs) using multivariable logistic regression analysis. Participants were divided into tertiles of dietary intake, with the lowest tertile considered as the reference group. In addition, the *p*-values for the trends were calculated using the median value of each tertile. Analysis of covariance (ANCOVA) with Bonferroni correction was performed to evaluate the mean differences in sarcopenia parameters among the tertiles after adjusting for confounding variables. All statistical analyses were performed using the SPSS software (version 27.0; SPSS Inc., Chicago, IL, USA); *p* < 0.05 was considered statistically significant.

## 3 Results

### 3.1 Characteristics of participants

The incidence of sarcopenia was 37.8% after the 6-year follow-up. Participants with sarcopenia were older with a lower BMI and higher proportion of cognitive impairment than participants without sarcopenia at the 6-year follow-up (Table 1). Other characteristics were not significantly different between sarcopenic and non-sarcopenic participants. Participants with sarcopenia consumed less oily fish than non-sarcopenic participants. However, there were no differences in the intake of seafood, fish, non-oily fish, or shellfish between the two groups.

**Table 1:** Baseline characteristics of the participants according to the incidence of sarcopenia after the 6-year follow-up.

**Variables**	**Follow-up (*n =* 503)**		***p*-value**
	**Non-sarcopenia (*****n** =* **313)**	**Sarcopenia (*****n** =* **190)**	
Age, years	75.28 ± 3.73	76.37 ± 3.96	0.003
Women, *n* (%)	172 (55.0)	95 (50.0)	0.281
Body mass index, kg/m^2^	25.23 ± 2.83	24.24 ± 2.63	< 0.001
Current smoker, *n* (%)	10 (3.2)	12 (6.3)	0.097
Education, years	8.88 ± 5.13	8.92 ± 5.31	0.983
Low economic status, *n* (%)	27 (8.6)	11 (5.8)	0.243
Living alone, *n* (%)	75 (24.0)	39 (20.5)	0.372
Number of comorbidities	1.73 ± 1.35	1.89 ± 1.35	0.190
Polypharmacy, *n* (%)	95 (30.4)	58 (30.5)	0.967
Sleep duration, *n* (%)			0.265
< 7 h	189 (60.4)	128 (67.4)	
7–8 h	107 (34.2)	52 (27.4)	
>8 h	17 (5.4)	10 (5.3)	
Fall in last year, *n* (%)	58 (18.5)	30 (15.8)	0.433
Fall-related fracture, *n* (%)	7 (2.2)	6 (3.2)	0.569
Low physical activity, *n* (%)	24 (7.7)	11 (5.8)	0.422
Cognitive impairment, *n* (%)	43 (13.7)	41 (21.6)	0.022
Nutritional status, *n* (%)			0.518
Normal	277 (88.5)	163 (85.8)	
At risk of malnutrition	35 (11.2)	26 (13.7)	
Malnutrition	1 (0.3)	1 (0.5)	
**Dietary intake**			
Energy intake, kcal	1,492.43 ± 432.28	1,447.85 ± 375.51	0.360
Seafood, g	54.27 ± 54.31	57.04 ± 60.73	0.886
Fish, g	39.53 ± 43.61	42.14 ± 47.54	0.873
Oily fish, g	16.17 ± 26.46	13.57 ± 23.89	0.021
Non-oily fish, g	23.35 ± 33.72	28.58 ± 41.23	0.538
Shellfish, g	14.74 ± 29.93	14.90 ± 28.51	0.864

Data are presented as mean ± SD or number of the participants (percentage distribution), as appropriate. p-values were analyzed using the independent t-test for parametric continuous variables, the Mann–Whitney U test for non-parametric continuous variables, and chi-squared test for categorical variables. SD, standard deviation.

### 3.2 Association between sarcopenia and intake of seafood

Multivariate logistic regression analysis revealed an inverse association between the incidence of sarcopenia and oily fish intake at the 6-year follow-up (Table 2). There was no significant association between the incidence of sarcopenia and intake of seafood, fish, non-oily fish, or shellfish.

**Table 2:** Logistic regression of seafood, fish, and shellfish intake for the incidence of sarcopenia after the 6-year follow-up.

	**Tertiles of food intake (g)**			***p* for trend**
	**T1**	**T2**	**T3**	
Seafood, g	≤ 19.50	19.50 < to ≤ 64.25	>64.25	
Sarcopenia/non-sarcopenia, *n*	64/104	60/108	66/101	
Adjusted OR (95% CI)	1.0	0.993 (0.619–1.591)	1.237 (0.772–1.983)	0.327
Fish, g	≤ 8.50	8.50 < to ≤ 45.50	> 45.50	
Sarcopenia/non-sarcopenia, n	66/102	57/111	67/100	
Adjusted OR (95% CI)	1.0	0.821 (0.512–1.316)	1.201 (0.751–1.921)	0.308
Oily fish, g	≤ 2.25	2.25 < to ≤ 9.75	>9.75	
Sarcopenia/non-sarcopenia, n	75/100	66/100	49/113	
Adjusted OR (95% CI)	1.0	0.942 (0.599–1.481)	0.635 (0.395–1.020)	0.046
Non-oily fish, g	0	0 < to ≤ 30.00	>30.00	
Sarcopenia/non-sarcopenia, n	92/148	28/70	70/95	
Adjusted OR (95% CI)	1.0	0.624 (0.367–1.063)	1.206 (0.785–1.853)	0.321
Shellfish, g	0	0 < to ≤ 8.80	>8.80	
Sarcopenia/non-sarcopenia, n	93/147	31/65	66/101	
Adjusted OR (95% CI)	1.0	0.819 (0.482–1.392)	1.228 (0.799–1.888)	0.249

Odds ratios (ORs) and 95% confidence intervals (CIs) are presented. The estimate of p for linear trends is based on the linear scores derived from the median of the tertiles of food intake among all participants. Adjusted OR and 95% CI were analyzed using logistic regression analysis after adjusting for age, body mass index, smoking status, living arrangements, number of comorbidities, cognitive impairment, fall experience, and sleep duration.

### 3.3 Associations between sarcopenia parameters and intake of seafood

In the multivariate model, the incidence of low UGS was inversely associated with oily fish intake (Table 3). Additionally, the highest tertile of oily fish intake was inversely associated with the incidence of low UGS compared to the lowest tertile after adjusting for confounding factors. Consistently, ANCOVA showed a significant positive association between UGS and oily fish intake after adjusting for confounding factors (Table 4). There were no significant associations between the other sarcopenia parameters and the intake of seafood, fish, non-oily fish, or shellfish (Supplementary Tables S1, S2).

**Table 3:** Logistic regression of seafood, fish, and oily fish intake for the incidence of each sarcopenia component after the 6-year follow-up.

	**Tertiles of food intake (g)**			***p* for trend**
	**T1**	**T2**	**T3**	
**Seafood**				
Low muscle mass, *n* (yes/no)	56/82	62/71	65/70	
Cut-off, g	≤ 19.50	19.50 < to ≤ 63.75	>63.75	
Adjusted OR (95% CI)	1.0	1.351 (0.804–2.270)	1.488 (0.887–2.494)	0.166
Low muscle strength, *n* (yes/no)	32/111	19/124	25/117	
Cut-off, g	≤ 19.75	19.75 < to ≤ 62.45	>62.45	
Adjusted OR (95% CI)	1.0	0.513 (0.266–0.989)	0.750 (0.401–1.401)	0.561
Low UGS, *n* (yes/no)	40/88	39/86	37/88	
Cut-off, g	≤ 20.00	20.00 < to ≤ 64.25	>64.25	
Adjusted OR (95% CI)	1.0	1.167 (0.662–2.058)	1.138 (0.640–2.025)	0.722
Low 5-STS score, *n* (yes/no)	41/76	34/82	39/76	
Cut-off, g	≤ 22.50	22.50 < to ≤ 69.00	>69.00	
Adjusted OR (95% CI)	1.0	0.854 (0.477–1.528)	1.155 (0.645–2.067)	0.522
Low SPPB score, *n* (yes/no)	25/112	21/116	27/109	
Cut-off, g	≤ 21.00	21.00 < to ≤ 65.40	>65.40	
Adjusted OR (95% CI)	1.0	0.883 (0.453–1.720)	1.390 (0.731–2.641)	0.242
**Fish**				
Low muscle mass, *n* (yes/no)	60/77	56/78	67/68	
Cut-off, g	≤ 8.25	8.25 < to ≤ 43.90	>43.90	
Adjusted OR (95% CI)	1.0	0.898 (0.534–1.509)	1.365 (0.817–2.280)	0.161
Low muscle strength, *n* (yes/no)	29/118	19/120	28/114	
Cut-off, g	≤ 9.00	9.00 < to ≤ 43.90	>43.90	
Adjusted OR (95% CI)	1.0	0.637 (0.328–1.240)	1.009 (0.543–1.873)	0.800
Low UGS, *n* (yes/no)	44/86	36/86	36/90	
Cut-off, g	≤ 9.00	9.00 < to ≤ 47.00	>47.00	
Adjusted OR (95% CI)	1.0	1.034 (0.585–1.826)	0.906 (0.516–1.593)	0.700
Low 5-STS score, *n* (yes/no)	40/76	31/85	43/73	
Cut-off, g	≤ 9.75	9.75 < to ≤ 49.38	>49.38	
Adjusted OR (95% CI)	1.0	0.824 (0.453–1.496)	1.348 (0.761–2.389)	0.233
Low SPPB score, *n* (yes/no)	26/111	20/117	27/109	
Cut-off, g	≤ 9.38	9.38 < to ≤ 49.38	>49.38	
Adjusted OR (95% CI)	1.0	0.953 (0.485–1.873)	1.348 (0.714–2.543)	0.310
**Oily fish**				
Low muscle mass, *n* (yes/no)	68/69	57/77	58/77	
Cut-off, g	≤ 2.00	2.00 < to ≤ 8.73	>8.73	
Adjusted OR (95% CI)	1.0	0.764 (0.460–1.266)	0.846 (0.511–1.402)	0.749
Low muscle strength, *n* (yes/no)	31/115	26/116	19/121	
Cut-off, g	≤ 2.25	2.25 < to ≤ 9.75	>9.75	
Adjusted OR (95% CI)	1.0	0.843 (0.458–1.551)	0.683 (0.355–1.313)	0.289
Low UGS, *n* (yes/no)	47/83	43/80	26/99	
Cut-off, g	≤ 2.50	2.50 < to ≤ 11.25	>11.25	
Adjusted OR (95% CI)	1.0	0.876 (0.507–1.513)	0.496 (0.277–0.891)	0.016
Low 5-STS score, *n* (yes/no)	43/74	36/80	35/80	
Cut-off, g	≤ 2.25	2.25 < to ≤ 11.25	>11.25	
Adjusted OR (95% CI)	1.0	0.754 (0.425–1.339)	0.861 (0.486–1.524)	0.859
Low SPPB score, *n* (yes/no)	31/110	23/111	19/116	
Cut-off, g	≤ 2.25	2.25 < to ≤ 10.50	>10.50	
Adjusted OR (95% CI)	1.0	0.727 (0.388–1.362)	0.654 (0.339–1.263)	0.308

Odds ratios (ORs) and 95% confidence intervals (CIs) are presented. The estimate of p for linear trends is based on the linear scores derived from the median of the tertiles of food intake among all participants. OR and 95% CI were analyzed using logistic regression analysis after adjusting for age, body mass index, smoking status, living arrangements, number of comorbidities, cognitive impairment, fall experience, and sleep duration. UGS, usual gait speed; 5-STS, 5-times sit-to-stand; SPPB, Short Physical Performance Battery.

**Table 4:** Sarcopenia parameters at the 6-year follow-up according to tertiles of seafood, fish, and oily fish intake.

	**Tertiles of food intake (g)**			***p*-value**
	**T1**	**T2**	**T3**	
**Seafood**				
Cut-off, g	≤ 19.50	19.50 < to ≤ 63.75	>63.75	
ASM index, kg/m^2^	6.18 ± 0.916	6.17 ± 0.920	6.37 ± 0.874	0.346
Cut-off, g	≤ 19.75	19.75 < to ≤ 62.45	>62.45	
Handgrip strength, kg	25.7 ± 7.24	26.4 ± 7.27	28.3 ± 7.63	0.076
Cut-off, g	≤ 20.00	20.00 < to ≤ 64.25	>64.25	
UGS, m/s	1.10 ± 0.224	1.10 ± 0.189	1.11 ± 0.210	0.753
Cut-off, g	≤ 22.50	22.50 < to ≤ 69.00	>69.00	
5-STS test, s	11.3 ± 4.17	10.9 ± 3.02	11.2 ± 3.33	0.669
Cut-off, g	≤ 21.00	21.00 < to ≤ 65.40	>65.40	
SPPB, score	11.0 ± 1.33	10.9 ± 1.29	10.9 ± 1.32	0.435
**Fish**				
Cut-off, g	≤ 8.25	8.25 < to ≤ 43.90	>43.90	
ASM index, kg/m^2^	6.22 ± 0.892	6.19 ± 0.927	6.32 ± 0.902	0.995
Cut-off, g	≤ 9.00	9.00 < to ≤ 43.90	>43.90	
Handgrip strength, kg	25.9 ± 7.15	26.7 ± 7.36	27.8 ± 7.77	0.608
Cut-off, g	≤ 9.00	9.00 < to ≤ 47.00	>47.00	
UGS, m/s	1.08 ± 0.209	1.12 ± 0.209	1.11 ± 0.204	0.787
Cut-off, g	≤ 9.75	9.75 < to ≤ 49.38	>49.38	
5-STS test, s	11.2 ± 3.41	10.7 ± 3.84	11.5 ± 3.31	0.230
Cut-off, g	≤ 9.38	9.38 < to ≤ 49.38	>49.38	
SPPB, score	10.9 ± 1.25	11.0 ± 1.40	10.8 ± 1.29	0.331
**Oily fish**				
Cut-off, g	≤ 2.00	2.00 < to ≤ 8.73	>8.73	
ASM index, kg/m^2^	6.17 ± 0.861	6.24 ± 0.933	6.32 ± 0.924	0.653
Cut-off, g	≤ 2.25	2.25 < to ≤ 9.75	>9.75	
Handgrip strength, kg	26.5 ± 7.71	26.0 ± 6.97	27.9 ± 7.56	0.413
Cut-off, g	≤ 2.50	2.50 < to ≤ 11.25	>11.25	
UGS, m/s	1.07 ± 0.184^a^	1.09 ± 0.219^a^	1.15 ± 0.211^b^	0.024
Cut-off, g	≤ 2.25	2.25 < to ≤ 11.25	>11.25	
5-STS test, s	11.5 ± 4.11	11.0 ± 3.09	10.9 ± 3.32	0.520
Cut-off, g	≤ 2.25	2.25 < to ≤ 10.50	>10.50	
SPPB, score	10.8 ± 1.44	10.9 ± 1.23	11.0 ± 1.24	0.323

Data are presented as mean ± SD, as appropriate. Adjusted p-values for the differences in sarcopenia parameters according to tertiles of food intake were assessed using ANCOVA with Bonferroni correction after adjusting for confounding factors, including age, body mass index, smoking status, living arrangement, number of comorbidities, cognitive impairment, fall experience, and sleep duration. Groups not sharing the same superscript letter (e.g., “a” vs. “b”) differ significantly (Bonferroni-adjusted p < 0.05). ASM, appendicular skeletal muscle mass; UGS, usual gait speed; 5-STS, 5-times sit-to-stand; SPPB, Short Physical Performance Battery; SD, standard deviation; ANCOVA, analysis of covariance.

## 4 Discussion

In the present study, the intake of oily fish, but not seafood or other fish, was significantly associated with the incidence of sarcopenia in community-dwelling older Koreans after a 6-year follow-up period. Consistently, the prevalence of sarcopenia is not associated with the frequency of fish and shrimp intake, which ranges from 0 to 7 times per week in older Chinese individuals (4–6). The average daily fish intake of approximately 40 g is similar to the 35 g reported in older Chinese individuals (34). However, the frequency of oily fish intake was reportedly five times higher among Korean adults (35) compared with Chinese adults (36). Although no study has investigated the association between oily fish intake and the incidence or prevalence of sarcopenia, previous studies have reported the positive association of the intake of oily fish, but not non-oily fish, with muscle strength and physical performance in older British (7, 8) and Spanish populations (9). The beneficial effects of oily fish on sarcopenia could be attributed to the higher content of n-3 PUFA compared to non-oily fish. Oily fish contain approximately seven times more n-3 PUFA, especially eicosapentaenoic acid (EPA) and docosahexaenoic acid (DHA), than non-oily fish (31). In the present study, the average daily intake of oily fish was approximately 15 g, similar to the positive associations between oily fish intake of 14–22 g and muscle strength (7) and physical performance (9). Furthermore, oily fish accounted for approximately 38% of the total fish intake in the present study, similar to the 39% reported in a Spanish study showing the beneficial effects of oily fish on physical performance (9). These previous studies support our finding that oily fish intake could affect sarcopenia, as achieving similar effects from non-oily fish would require substantially higher consumption.

Consistently, the intake of n-3 PUFA has been inversely associated with the prevalence of sarcopenia in older Australian men (10), Japanese individuals with type 2 diabetes (11), and Brazilian recipients of kidney transplant (12). Yang et al. (16) reported that the ratio of n-3 PUFA to energy intake was inversely associated with the prevalence of sarcopenic obesity in older Korean women based on data from KNHANES. Meta-analyses of clinical studies have indicated that supplementation of n-3 PUFA improves muscle mass, muscle strength, and physical performance in older adults (23, 24). In immobilized aged rodent models, n-3 PUFA reportedly increased muscle protein synthesis by activating the mTOR pathway, enhancing amino acid transport, and decreased muscle protein breakdown by reducing inflammatory signaling and inhibiting the ubiquitin-proteasome system (25).

Blood levels of n-3 PUFA, an objective biomarker of the dietary intake of n-3 PUFA, were inversely associated with the prevalence of sarcopenia in older Koreans (17) and Japanese patients with liver cirrhosis and hepatocellular carcinoma (18), but not in older Dutch adults (37). The erythrocyte levels of EPA and DHA were approximately 9% in Koreans and Japanese (38), but 4% in Dutch individuals (38). Additionally, the prevalence rate of sarcopenia was reportedly 13–20% in older Koreans and Japanese (39), but 22–32% in older Dutch individuals (40–42), which is higher than that in Asians. In the present study, the incidence of sarcopenia was 37.8% after the 6-year follow-up among older Koreans, aligning with the annual incidence estimate of 6.3% reported by the KFACS study (43).

In the present study, the consumption of oily fish, but not seafood or fish, was inversely associated with the incidence of low UGS at the 6-year follow-up. Our previous study also found that the consumption of oily fish was inversely associated with the prevalence of low UGS in older British individuals based on data from the UK Biobank (15). However, no significant association was observed between the intake of oily fish and UGS in older British individuals in the Hertfordshire Cohort Study (44). The intake of oily fish was similar in older British adults in the UK Biobank study and Hertfordshire Cohort Study (44, 45). However, the average UGS was 1.1 m/s among older British based on the UK Biobank (46), and 0.9 m/s from the Hertfordshire Cohort Study (44), suggesting that older British in Hertfordshire Cohort had slower UGS than those from UK Biobank. In the present study, the average UGS was 1.1 m/s, which is consistent with a previous KFACS study (47). Furthermore, n-3 PUFA intake has been positively associated with UGS in older Finnish women (13), Australian individuals with subjective memory complaints (14), and older British adults (15). Consistently, blood levels of n-3 PUFA have been positively associated with UGS in older British (15), French (19), Italian (20), Korean (21), and Japanese populations (22). A meta-analysis of clinical studies also showed that n-3 PUFA supplementation improved UGS in older Americans and Europeans (24).

There were no associations in the present study between seafood, fish, and oily fish consumption and muscle mass, handgrip strength, 5-STS test, and SPPB score. Previous studies consistently reported that the consumption of seafood or fish was not associated with muscle mass in older Chinese (48) and British women (49); handgrip strength in older Koreans (50), Norwegians (51), Finns (52), and Italian women (53); and the 5-STS test in older British individuals (44). Furthermore, the SPPB score was not associated with seafood consumption in older Norwegians (51) or Australian individuals with type 2 diabetes (54). Regarding oily fish consumption, there was no previous study investigating the association with muscle mass. Consistent with the present study, Martin et al. (44) also found no association between oily fish intake and 5-STS test in older British individuals. In the present study, there was no significant association between oily fish intake and handgrip strength, but the positive association was reported in older British individuals (7, 8, 15). These discrepancies could be due to the method of measuring handgrip strength, since the present study used a digital dynamometer but other studies used a hydraulic dynamometer. Savas et al. (55) reported that handgrip strength measured using a hydraulic dynamometer was lower than that measured using a digital dynamometer in adults, particularly in those >60 years of age. Unlike the present study, previous studies reported a positive association between oily fish intake and the SPPB score in Spanish population (9, 56). There were racial differences between the present study and the previous studies, and the cut-off score SPPB was also different; 6 in the previous studies and 9 in the present study. The cut-off score for SPPB is 9 according to AWGS (27), while 8 according to the European Working Group on Sarcopenia (57).

This is the first longitudinal study to show that the intake of oily fish, but not seafood or other fish, is inversely associated with the incidence of sarcopenia among older adults after a 6-year follow-up. However, this study had a few limitations. First, dietary data were obtained using two non-consecutive days of 24-h dietary recalls during two different seasons, which might not be sufficient to reflect the usual dietary intake. Second, although potential confounders were adjusted for in the analysis, residual confounding factors such as n-3 PUFA supplementation and other dietary influences may remain. Third, cooking methods such as frying or roasting could reduce the content of n-3 PUFA due to increased oxidation (58), but the study did not account for these cooking methods. Last, our findings were obtained only from Korean older adults, which might not be generalized to other populations.

## 5 Conclusion

The consumption of oily fish could have beneficial effects in preventing sarcopenia, partly by improving walking speed in community-dwelling older Koreans, suggesting oily fish as a strategy to reduce sarcopenia risk. Further clinical studies are required to confirm the preventive effects of n-3 PUFA against sarcopenia.

## Data Availability

The original contributions presented in the study are included in the article/Supplementary material, further inquiries can be directed to the corresponding author.
