# Supplementary material for: Association between fish intake and incidence of sarcopenia in community-dwelling older adults after a 6-year follow-up: the Korean frailty and aging cohort study
Source: Front Nutr. 2025 Jan 28;12:1543290. doi: 10.3389/fnut.2025.1543290 (PMC11810723; doi:10.3389/fnut.2025.1543290)
Supplement: Supplementary file 1 [file Table_1.docx]

Supplementary Material

# Supplementary Tables

**Table S1.** Logistic regression of non-oily fish and shellfish intake for the incidence of each sarcopenia component after the 6-year follow-up

|  | Tertiles of food intake (g) | | | *p* for trend |
| --- | --- | --- | --- | --- |
|  | T1 | T2 | T3 |  |
| Non-oily fish |  |  |  |  |
| Low muscle mass, n (yes/no) | 85/107 | 32/49 | 66/67 |  |
| Cut-off, g | 0 | 0 < to ≤ 30.00 | > 30.00 |  |
| Adjusted OR (95% CI) | 1.0 | 0.772 (0.443–1.347) | 1.232 (0.767–1.980) | 0.344 |
| Low muscle strength, n (yes/no) | 36/168 | 12/72 | 28/112 |  |
| Cut-off, g | 0 | 0 < to ≤ 30.00 | > 30.00 |  |
| Adjusted OR (95% CI) | 1.0 | 0.758 (0.363–1.583) | 1.027 (0.574–1.837) | 0.878 |
| Low UGS, n (yes/no) | 60/121 | 18/55 | 38/86 |  |
| Cut-off, g | 0 | 0 < to ≤ 30.00 | > 30.00 |  |
| Adjusted OR (95% CI) | 1.0 | 0.665 (0.347–1.274) | 0.956 (0.571–1.601) | 0.966 |
| Low 5-STS score, n (yes/no) | 53/108 | 21/50 | 40/76 |  |
| Cut-off, g | 0 | 0 < to ≤ 30.00 | > 30.00 |  |
| Adjusted OR (95% CI) | 1.0 | 0.907 (0.482–1.708) | 1.152 (0.676–1.963) | 0.584 |
| Low SPPB score, n (yes/no) | 33/160 | 19/93 | 21/84 |  |
| Cut-off, g | 0 | 0 < to ≤ 35.00 | > 35.00 |  |
| Adjusted OR (95% CI) | 1.0 | 1.174 (0.612–2.252) | 1.389 (0.736–2.623) | 0.314 |
| Shellfish |  |  |  |  |
| Low muscle mass, n (yes/no) | 89/104 | 32/46 | 62/73 |  |
| Cut-off, g | 0 | 0 < to ≤ 9.38 | > 9.38 |  |
| Adjusted OR (95% CI) | 1.0 | 0.815 (0.463–1.436) | 1.055 (0.659–1.690) | 0.689 |
| Low muscle strength, n (yes/no) | 41/163 | 7/76 | 28/113 |  |
| Cut-off, g | 0 | 0 < to ≤ 8.75 | > 8.75 |  |
| Adjusted OR (95% CI) | 1.0 | 0.381 (0.157–0.923) | 1.116 (0.630–1.980) | 0.443 |
| Low UGS, n (yes/no) | 52/124 | 26/51 | 38/87 |  |
| Cut-off, g | 0 | 0 < to ≤ 8.75 | > 8.75 |  |
| Adjusted OR (95% CI) | 1.0 | 1.386 (0.749–2.565) | 1.234 (0.724–2.105) | 0.577 |
| Low 5-STS score, n (yes/no) | 58/95 | 24/60 | 32/79 |  |
| Cut-off, g | 0 | 0 < to ≤ 10.00 | > 10.00 |  |
| Adjusted OR (95% CI) | 1.0 | 0.752 (0.413–1.369) | 0.684 (0.394–1.188) | 0.236 |
| Low SPPB score, n (yes/no) | 39/151 | 11/73 | 23/113 |  |
| Cut-off, g | 0 | 0 < to ≤ 8.80 | > 8.80 |  |
| Adjusted OR (95% CI) | 1.0 | 0.593 (0.277–1.269) | 0.919 (0.505–1.670) | 0.993 |

Odds ratios (ORs) and 95% confidence intervals (CIs) are presented. The estimate of *p* for linear trends is based on the linear scores derived from the median of the tertiles of food intake among all participants. OR and 95% CI were analyzed using logistic regression analysis after adjusting for age, body mass index, smoking status, living arrangements, number of comorbidities, cognitive impairment, fall experience, and sleep duration. UGS, usual gait speed; 5-STS, 5-times sit-to-stand; SPPB, Short Physical Performance Battery.

**Table S2.** Sarcopenia parameters at the 6-year follow-up according to tertiles of non-oily fish and shellfish intake

|  | Tertiles of food intake (g) | | | *p*-value |
| --- | --- | --- | --- | --- |
|  | T1 | T2 | T3 |  |
| Non-oily fish |  |  |  |  |
| Cut-off, g | 0 | 0 < to ≤ 30.00 | > 30.00 |  |
| ASM index, kg/m^2^ | 6.22 ± 0.913 | 6.18 ± 0.940 | 6.32 ± 0.877 | 0.929 |
| Cut-off, g | 0 | 0 < to ≤ 30.00 | > 30.00 |  |
| Handgrip strength, kg | 26.3 ± 7.31 | 26.6 ± 7.83 | 27.5 ± 7.41 | 0.612 |
| Cut-off, g | 0 | 0 < to ≤ 30.00 | > 30.00 |  |
| UGS, m/s | 1.10 ± 0.212 | 1.14 ± 0.197 | 1.09 ± 0.206 | 0.127 |
| Cut-off, g | 0 | 0 < to ≤ 30.00 | > 30.00 |  |
| 5-STS test, s | 11.1 ± 3.62 | 11.0 ± 4.31 | 11.2 ± 2.86 | 0.904 |
| Cut-off, g | 0 | 0 < to ≤ 35.00 | > 35.00 |  |
| SPPB, score | 11.0 ± 1.28 | 10.9 ± 1.32 | 10.8 ± 1.37 | 0.533 |
| Shellfish |  |  |  |  |
| Cut-off, g | 0 | 0 < to ≤ 9.38 | > 9.38 |  |
| ASM index, kg/m^2^ | 6.20 ± 0.894 | 6.35 ± 0.978 | 6.25 ± 0.881 | 0.463 |
| Cut-off, g | 0 | 0 < to ≤ 8.75 | > 8.75 |  |
| Handgrip strength, kg | 26.2 ± 7.59 | 27.9 ± 7.43 | 27.0 ± 7.21 | 0.290 |
| Cut-off, g | 0 | 0 < to ≤ 8.75 | > 8.75 |  |
| UGS, m/s | 1.10 ± 0.213 | 1.10 ± 0.212 | 1.11 ± 0.198 | 0.970 |
| Cut-off, g | 0 | 0 < to ≤ 10.00 | > 10.00 |  |
| 5-STS test, s | 11.5 ± 4.18 | 10.8 ± 2.75 | 10.9 ± 3.03 | 0.340 |
| Cut-off, g | 0 | 0 < to ≤ 8.80 | > 8.80 |  |
| SPPB, score | 10.8 ± 1.36 | 11.0 ± 1.20 | 11.0 ± 1.31 | 0.475 |

Data are presented as mean ± SD, as appropriate. Adjusted *p*-values for the differences in sarcopenia parameters according to tertiles of food intake were assessed using ANCOVA with Bonferroni correction after adjusting for confounding factors, including age, body mass index, smoking status, living arrangement, number of comorbidities, cognitive impairment, fall experience, and sleep duration. Groups not sharing the same superscript letter (e.g., “a” vs. “b”) differ significantly (Bonferroni-adjusted *p* < 0.05). ASM, appendicular skeletal muscle mass; UGS, usual gait speed; 5-STS, 5-times sit-to-stand; SPPB, Short Physical Performance Battery; SD, standard deviation; ANCOVA, analysis of covariance.
